# Use of the confusion assessment method in multicentre delirium trials: training and standardisation

**DOI:** 10.1186/s12877-019-1129-8

**Published:** 2019-04-15

**Authors:** John R. Green, Jane Smith, Elizabeth Teale, Michelle Collinson, Michael S. Avidan, Eva M. Schmitt, Sharon K. Inouye, John Young

**Affiliations:** 1 Academic Unit of Elderly Care and Rehabilitation, Bradford Institute for Health Research Temple Bank House, Bradford Royal Infirmary, Duckworth Lane, Bradford, BD96RJ UK; 2 Clinical Trials Research Unit, Leeds Institute of Clinical Trials Research, University of Leeds, Leeds, UK; 3 Washington University School of Medicine, 660 S. Euclid Ave, St. Louis, MO 63110 USA; 4 Institute for Aging Research Hebrew SeniorLife and Harvard Medical School, 1200 Centre Street, Boston, MA 02131 USA

**Keywords:** Delirium, Confusion assessment method, Multicentre studies, Training

## Abstract

**Background:**

Delirium occurs commonly in older adults and is associated with adverse outcomes. Multicentre clinical trials evaluating interventions to prevent delirium are needed. The Confusion Assessment Method (CAM) is a validated instrument for delirium detection. We hypothesised it would be possible for a large feasibility study to train a large number of research assistants, with varying experience levels, to conduct CAM assessments reliably in multiple hospital sites.

**Methods:**

A standardised training programme was followed, incorporating structured training at a central location and at study sites. CAM practice sessions on both delirious and non-delirious patients by research assistants were conducted and, thereafter, there was ongoing inter-rater reliability assessment on the CAM between research assistant pairs at study sites. The setting was eight acute care hospitals in England and Wales. Participants were research assistants working on a multicentre feasibility study of delirium prevention. The measurement used was the Confusion Assessment Method.

**Results:**

Thirty-seven research assistants were trained in CAM assessment and 33 returned training logs. The logs showed there was 100% overall agreement between research assistant pairs on 295 CAM assessments, of which 263 (89.2%) were negative for delirium and 32 (10.8%) were positive. In the course of the feasibility study, research assistants successfully completed 5065 (89.7%) of the 5645 expected CAM assessments, with minimal missing data.

**Conclusion:**

Using the training methods described in this study, it is possible to achieve high quality delirium assessments for large numbers of patients with little missing data across geographically dispersed sites in multicentre studies. The standardisation of multisite delirium assessments is an important contribution to research methodology, and provides a much-needed advance for the field.

**Trial registration:**

ISRCT ISRCTN01187372. Registered 13 March 2014.

## Background

Delirium occurs commonly in hospitalised older people [1] and is associated with adverse outcomes [2]. The features of delirium are described in the Diagnostic and Statistical Manual of Mental Disorder and have been operationalised in a range of delirium screening and detection tools [3]. However, the clinical assessment of these features of delirium may not be straightforward due to symptom fluctuation and/or difficulty in distinguishing delirium from other conditions such as chronic cognitive impairment, dementia or depression. The choice of instrument depends on the purpose of assessment, the time available for completion, and experience of the assessor. The Confusion Assessment Method (CAM) [4] has been widely used in clinical practice and in research studies [5]. Administration of the CAM typically takes 5–10 min and is informed by brief, formal cognitive assessment [6]. Validation studies have reported high sensitivity (94–100%) and specificity (90–95%) in the hands of clinicians or researchers trained in its use [3, 7]. Robust adherence to the processes described in the training manual is recommended to optimise diagnostic accuracy.

The number of multisite clinical trials for delirium are increasing greatly driving the need for feasible and effective approaches to standardise ratings across sites. In a systematic review of clinical trials for delirium prevention published in 2016, six multicentre clinical trials were included [1]. A recent search of ClinicalTrials.gov (20 July 2018) yielded 28 active or completed multicentre clinical trials. We report here the methods developed to achieve the recruitment and training of a large number of research assistants (RAs) in a multisite cluster randomised trial of delirium prevention [8]. A key outcome of the study was incident delirium measured with the CAM. This required the recruitment and training of a large number of RAs. We hypothesised that it would be feasible to train a large number of RAs, with varying experience levels, to complete CAM assessments reliably across all study sites. We also provide information on completion rates of the CAM in the trial. This study is unique and innovative in being one of the first to conduct a large-scale study of this nature addressing standardisation of CAM ratings across many sites.

## Methods

The Prevention of Delirium research programme aimed to develop and test a non-pharmacological, multicomponent intervention to reduce the incidence of delirium in older people admitted to hospital [8]. Preliminary testing of the intervention was via a cluster randomised, multicentre feasibility study involving 713 participants from 16 acute care of the elderly and surgical/trauma orthopaedic wards in eight acute care hospitals in England and Wales [8, 9]. Study sites were randomised in February 2014, after which sites initiated a six-month intervention implementation period. Patient screening and recruitment took place between August 2014 and February 2015. Patients were eligible if they were aged 65 years or older and had no delirium when admitted to the study wards [8]. Study participants had a mean age (standard deviation) of 82.7 (7.84) years; 170 (23.8%) had been admitted with a hip fracture, 162 (22.7%) with another orthopaedic condition, and 380 (53.3%) with a medical condition; 150 (21.0%) had cognitive impairment or dementia; 113 (15.8%) were severely ill; 232 (32.5%) had a hearing impairment and 634 (88.9%) had a visual impairment. Data collection was completed in August 2015. Full details of the study methods are reported elsewhere [8]. Patients consenting to the study were screened daily for delirium by RAs using the CAM for up to 10 days whilst in hospital. Each of the eight participating NHS trusts received funding for up to two full time equivalent RAs.

### Training procedures

Following the RA appointments, CAM training and monitoring took place in three stages. Stage 1 was classroom teaching about study research procedures, delirium, and the administration of outcome measurement instruments including the CAM. Stage 2 was CAM-specific and involved local experiential learning consisting of a) one-to-one practice sessions, b) pilot interviews with patients and c) within-site inter-rater reliability (IRR) assessments. Stage 3 was a further within-site IRR CAM performance check conducted during the feasibility study at local sites.

#### Stage 1: central or local classroom teaching

Training was offered either centrally or locally depending on the preference of each site and delivered by researchers from the coordinating centre (the Academic Unit of Elderly Care and Rehabilitation). The content of the training was the same for central and local sessions. Classroom teaching lasted for approximately seven to eight hours for both central and local sessions. Each RA was provided with a personal copy of the CAM manual [6] and a detailed overview of the CAM was presented followed by in-depth guidance and discussion structured around the CAM Case Report Form developed for the study. We used video clips featuring actors to illustrate the bedside assessment process for the key features of delirium (inattention, disorganised thinking and altered level of consciousness) (Table 1).

**Table 1:** Structured assessment process to complete the Confusion Assessment Method

CAM Item	Source of information
1. Acute onset and fluctuating course	• Ward staff or relative/carer who knows the patient’s baseline mental status and has observed the patient over time. Inspection of the medical and nursing records. • Previous assessments
2. Inattention	• Informal general conversation • Formal cognitive testing: abbreviated mental test [10]); Months of the Year Backwards test [11]
3. Disorganised thinking	• Informal general conversation • Observations during completion of the abbreviated mental test score [10] and Months of the Year Backwards test [11]
4. Altered level of consciousness	• Information from ward staff • Informal general conversation • Bedside observation

The CAM can be accessed at: www.HospitalElderLifeProgram.org [12]

*CAM* confusion assessment method

To consolidate learning, we presented additional video clips of actors supplemented by background fictional clinical details and asked the RAs to identify the features of delirium exhibited. The training videos used in the study are not publically available; other examples of training videos can be found on the Hospital Elder Life Program website [12]. We provided instruction on the standardised procedure to be used when performing CAM assessments [6]. Firstly, RAs were to collect relevant information about the baseline cognitive status of the patient by interview from ward staff and/or relatives/carers who knew the patient’s baseline mental status and had observed the patient over time. Following a general conversation with the patient, formal cognitive testing was to be undertaken using the abbreviated mental test score (AMTS) [10] and the Months of the Year Backwards (MotYB) test [11]. Finally, the CAM was to be scored. Guided role play during which the RAs took turns at playing the patient or researcher was used to provide an opportunity to practice the CAM assessment.

#### Stage 2: local experiential learning

After the initial training, and in accordance with the recommended CAM training procedures [6], the RAs conducted local one-to-one practice sessions with colleagues, pilot interviews with patients and IRR assessments with local colleagues [6]. The one-to-one practice sessions required pairs of RAs to conduct interviews with one another to familiarise themselves with the content and procedure of undertaking the CAM assessment. Following these practice sessions, RAs carried out pilot interviews in pairs with delirious and non-delirious ward patients, with a recommended training goal of two with delirium and two without. Patients for pilot interviews were identified by senior medical staff. Vulnerable patients were approached by RAs following their or their relatives’ informal agreement obtained by senior nursing or medical ward staff. IRR assessments were then undertaken. During these paired assessments, one RA administered the cognitive assessment and CAM and the other observed. Both RAs then independently scored the CAM assessment. Roles were reversed in the next paired interview. The CAM training manual recommends this process is repeated on at least five delirious and five non-delirious patients until 100% agreement is achieved. The pilot interviews and IRR assessments were undertaken on inpatients after obtaining verbal agreement as part of the CAM training process for the RAs and before the start of patient enrolment into the feasibility study.

#### Stage 3. CAM performance check

During the feasibility study, RAs were requested to repeat IRR assessments after their site had been screening participants for four months.

The RAs were asked to keep personal training logs of CAM practice sessions, pilot interviews and all IRR assessments including the performance check. This information was used to assess the extent of the adherence to the CAM training processes.

### CAM assessments during the multicentre feasibility study

During the multicentre study, each CAM item was assessed and recorded on a dated and signed Clinical Research Form which was subsequently entered into a study-specific database ready for analysis.

## Results

Thirty seven RAs were employed to work in the eight study sites (Table 2). The median (interquartile range (IQR); range) number of RAs per site was 4.5 (IQR 2.5–6.5; 2–8). Due mainly to staff movement, the number of RAs working on the study fluctuated in most sites during the course of the study. The RAs in two sites worked exclusively on the present study; in the other sites, the RAs were part of clinical research networks and may have also been working on other studies. Several of the RAs worked exclusively at weekends. The RAs varied in their level of research experience between very experienced and having no previous research experience but none had been involved with delirium research before. The experienced RAs, as identified by the individual sites, assisted with training, mentoring and oversight of the more inexperienced RAs as available at the individual sites. As the study progressed, these more senior researchers changed their role to one of monitoring, support and absence coverage.

**Table 2:** Experiential training of the research assistants with the Confusion Assessment Method

Site	RA	CAM experiential training procedures						
		One-to-one sessions (N)	Pilot interviews with patients (N)			Inter-rater reliability assessments (N)		
			N	No delirium	Delirium	N	No delirium	Delirium
1	1	6	4	1	3	10	5	5
	2	6	4	1	3	10	5	5
2	3	0	4	4	0	6	6	0
	4	0	–	–	–	7	7	0
	5	0	4	3	1	5	5	0
	6	0	0	–	–	9	9	0
3	7	2	4	4	0	19	18	1
	8	4	4	4	0	20	19	1
	9	4	4	4	0	9	9	0
	10	4	4	4	0	12	12	0
	11	3	4	4	0	10	10	0
4	12	0	3	3	0	13	12	1
	13	3	4	3	1	10	9	1
	14	0	2	2	0	10	10	0
	15	–	–	–	–	–	–	–
	16	1	2	2	0	5	5	0
	17	6	3	3	0	6	6	0
	18	1	4	4	0	2	2	0
5	19	–	–	–	–	–	–	–
	20	2	3	3	0	6	6	0
	21	2	3	2	1	6	6	0
	22	–	–	–	–	–	–	–
	23	–	–	–	–	–	–	–
6	24	2	0	–	–	25	22	3
	25	2	0	–	–	13	11	2
	26	1	0	–	–	5	5	0
	27	2	0	–	–	4	4	0
	28	1	0	–	–	4	4	0
	29	1	0	–	–	6	5	1
	30	1	0	–	–	3	3	0
	31	1	0	–	–	12	10	2
7	32	2	1	1	0	7	4	3
	33	2	1	1	0	7	4	3
	34	2	1	1	0	7	5	2
	35	2	1	1	0	7	5	2
8	36	3	4	4	0	10	10	0
	37	3	4	4	0	10	10	0
Total		69	72	63	9	295^a^	263	32
Mean (SD)		2.1 (1.72)	2.3 (1.74)			8.9 (4.94)		
Median (IQR)		2 (1–3)	3 (0–4)			7 (6–10)		
Range		0–6	0–4			2–25		

*RA* research assistant, *CAM* confusion assessment method, *SD* standard deviation, *IQR* interquartile range

^a^139 patient assessments were 1:1 (interviewer:observer); 3 were 1:2; 2 were 1:3

### Stage 1: central or local classroom teaching

Fifteen RAs from six sites were trained in the two-day central training session; 24 from four sites were trained in their local sites; three attended both the central and local training sessions; one was trained locally by a senior research nurse at the site. The type of training received was determined based on staff availability, initiation of employment contracts, and local circumstances. While the central training (or combination of central plus local) was preferred, many sites were not able to complete this due to timing of contracts or other local circumstances. All the stipulated goals of training were achieved at each of these sessions.

Thirty six (97.3%) of the 37 RAs completed CAM training logs; however three logs from one site were unobtainable. One of the RAs joined the study at a later stage and was already experienced in delirium and CAM assessment.

### Stage 2: local experiential learning

Nineteen (57.6%) of the 33 RAs who returned the training log completed all recommended sections of training, i.e. one-to-one sessions, pilot interviews and IRR assessments. Five (15.2%) did not undertake one-to-one sessions; eight (24.2%) did not undertake pilot interviews with patients; and one (3.0%) did not undertake a one-to-one session or pilot interviews with patients. All 33 RAs undertook IRR assessments (median: 7; range 2–25) (Table 2) with 100% agreement between pairs of assessors for each assessment. CAM assessments were undertaken on 144 patients: of the 295 CAM ratings by the RAs, 263 (89.2%) scored negative for delirium and 32 (10.8%) scored positive (Table 2).

### Stage 3: CAM performance check

Twenty three (62.2%) of the 37 RAs undertook performance check IRR assessments (*n* = 90 on 45 patients; median 3; range 2–12 assessments). Of the 14 who did not: seven no longer worked on the study; two had already completed screening and recruitment; two worked only at weekends and check assessments were missing for three. Of the 45 assessments, 42 (93.3%) were scored negative and three (6.7%) scored positive for delirium. IRR agreement between the pairs of RAs for each assessment was 100%.

### CAM assessments undertaken in the multicentre feasibility study

Five thousand and sixty-five (89.7%) of the 5645 expected CAM assessments were undertaken by the RAs within the first 10 days of ward admission (Table 3). Non-completion rates ranged from 3.5 to 14.8% (Table 3). The main reasons for non-completion of the CAM were participants were too ill or participant refusal.

**Table 3:** Number (%) of in-hospital Confusion Assessment Method assessments performed and delirium incidence

	Site								Total
	1	2	3	4	5	6	7	8	
CAMs performed^a^									
Number expected^b^	862	639	824	672	586	506	668	888	5645
Number performed	734 (85.2%)	564 (88.3%)	795 (96.5%)	599 (89.1%)	523 (89.2%)	443 (87.5%)	611 (91.5%)	796 (89.6%)	5065 (89.7%)
AMTS performed	734 (100.0%)	564 (100.0%)	795 (100.0%)	598 (99.8%)	523 (100.0%)	443 (100.0%)	611 (100.0%)	795 (99.9%)	5063 (100%)
MotYB test performed	733 (99.9%)	563 (99.8%)	790 (99.4%)	596 (99.5%)	517 (98.9%)	441 (99.5%)	609 (99.7%)	791 (99.4%)	5040 (99.5%)
CAMs not performed									
Number not performed:	128 (14.8%)	75 (11.7%)	29 (3.5%)	73 (10.9%)	63 (10.8%)	63 (12.5%)	57 (8.5%)	92 (10.4%)	580 (10.3%)
-Participant too ill	42 (32.8%)	17 (22.7%)	14 (48.3%)	9 (12.3%)	25 (39.7%)	16 (25.4%)	22 (38.6%)	41 (44.6%)	186 (32.1%)
-Participant refused	13 (10.2%)	32 (42.7%)	9 (31.0%)	21 (28.8%)	22 (34.9%)	36 (57.1%)	17 (29.8%)	13 (14.1%)	163 (28.1%)
-Personal or nominated consultee refused	0 (0.0%)	1 (1.3%)	0 (0.0%)	0 (0.0%)	0 (0.0%)	2 (3.2%)	0 (0.0%)	0 (0.0%)	3 (0.5%)
-Participant unavailable	6 (4.7%)	14 (18.7%)	5 (17.2%)	26 (35.6%)	9 (14.3%)	7 (11.1%)	14 (24.6%)	25 (27.2%)	106 (18.3%)
-Research staff missed participant	0 (0.0%)	9 (12.0%)	1 (3.4%)	8 (11.0%)	6 (9.5%)	1 (1.6%)	2 (3.5%)	2 (2.2%)	29 (5.0%)
-Ward closed	61 (47.7%)	0 (0.0%)	0 (0.0%)	0 (0.0%)	0 (0.0%)	0 (0.0%)	0 (0.0%)	0 (0.0%)	61 (10.5%)
-Other	2 (1.6%)	2 (2.7%)	0 (0.0%)	8 (11.0%)	1 (1.6%)	1 (1.6%)	2 (3.5%)	0 (0.0%)	16 (2.8%)
-Missing	4 (3.1%)	0 (0.0%)	0 (0.0%)	1 (1.4%)	0 (0.0%)	0 (0.0%)	0 (0.0%)	11 (12.0%)	16 (2.8%)
Participant CAM results									
Positive – delirium suggested (N,%)	5 (4.8%)	5 (6.1%)	9 (8.6%)	9 (10.0%)	9 (12.9%)	3 (4.6%)	6 (6.5%)	11 (10.6%)	57 (8.0%)
Negative – delirium not suggested (N,%)	99 (95.2%)	77 (93.9%)	96 (91.4%)	81 (90.0%)	61 (87.1%)	62 (95.4%)	87 (93.5%)	93 (89.4%)	656 (92.0%)
Incidence (95%CI)	4.8 (0.70, 8.92)	6.1 (0.92, 11.28)	8.6 (3.22, 13.93)	10.0 (3.80, 16.20)	12.9 (5.02, 20.70)	4.6 (0.00, 9.72)	6.5 (1.46, 11.44)	10.6 (4.67, 16.49)	8.0 (6.00, 9.99)

*CAM* confusion assessment method, *AMTS* abbreviated mental test score, *MotYB* months of the year backwards test

^a^Figures relate to 712 patients as 1 patient withdrew at baseline and no further data was provided. ^b^The number expected excludes assessments unavailable due to discharge, death or withdrawal

Of the 5065 CAM assessments, six (0.1%) had missing responses to CAM questions, two (0.04%) omitted the AMTS and 25 (0.5%) omitted the MotYB test (Table 3). Fifty seven (8.0%) of the 713 participants developed new onset delirium within 10 days of ward admission. Delirium incidence between sites ranged from 4.6 to 12.9% (Table 3).

## Discussion

### Strengths

The CAM is quick to conduct, widely used, well tolerated by older adults, and can be rated by non-specialists [13] but optimal use requires ongoing training and practice for CAM assessors. This study demonstrates that it is feasible to train a large number of RAs, without prior experience in delirium research, to conduct a large number of delirium assessments. Only 10.3% of expected delirium assessments were not conducted, and the CAM interviews had minimal missing data. The robust methodology presented here will be useful to researchers and funders planning future multicentre trials focused on delirium prevention. A major strength was that this was an innovative study in being one of the first to undertake a large-scale standardisation process for the CAM.

### Limitations

There are several important limitations to be noted and lessons learned relating to the training and standardisation process. Firstly, the training involved considerable input from the coordinating research team to ensure all the RAs were trained in the CAM (and study procedures) to the same standard, and included organisation and delivery of a central training event and travel to five of the eight sites to deliver local training. This was anticipated but nonetheless time-consuming. It will need careful preparation and allocation of sufficient research resources for future multicentre delirium studies. Secondly, not all of the RAs undertook all of the elements of the recommended CAM practice and less than half achieved the recommended number of patient sessions (Table 2). Thirdly, the majority of RAs undertook IRR assessments (median 7) with site colleagues, yet only 14 (42.4%) carried out the recommended 10 (or more) IRR assessments. Moreover, even fewer RAs undertook the four-month performance check IRRs and there were no cross-site reliability checks. Finally, RA experience of patients with delirium was not extensive during the practice and IRR CAMs: only a small number of the training and IRR CAM assessments were positive (Table 2). Thus, only 16 (48.5%) of the 33 RAs assessed a patient with delirium during the training process. Given the large number of sites and raters, we could not assure that every rater was trained by a reference-standard CAM assessor. Thus, while IRR was assessed across all sites, we were unable to assess accuracy of every rater across all sites. It is possible that some aspects of the training were more important than others; however, our design did not allow us to examine this. An important aspect to consider is that an important advantage of the longer training is the opportunity for the RAs to practice and gain confidence.

Due to the geographical dispersal of the sites, the coordinating centre researchers were not able to supervise directly the Stage 2 training. We were therefore unable to witness how RAs actually performed the CAM and there is uncertainty concerning the conduct of some of the logged assessments. There was 100% agreement noted in the logs for the paired CAM assessments, which may indicate that the interviewer and observer assessments were not truly independent. However, there is also an increased likelihood of high agreement if most of the assessments are negative. Since only 32 (10.8%) of the 295 IRR ratings were CAM positive, this is likely to have been the case. The organisation of multicentre research studies in England and Wales meant that the RAs working on the feasibility trial were employed by the local sites, not the coordinating centre. The study investigators therefore had neither involvement in the hiring of the RAs (except at one site) to assure appropriate experience, nor day-to-day supervisory authority and thus had limited input into ongoing monitoring. The differing number of RAs between sites and their research background and experience could have influenced the consistency of the CAM assessments. Although we demonstrated excellent within-site reliability in CAM assessment, our study was not designed to assess between-site reliability. The large number of RAs between sites and their differing levels of research background and experiences made the detailed training and standardisation process very important for this study and other studies of this type.

Non-completion rates of the CAM were low and mostly due to participants becoming too unwell to assess. These participants may have been more likely to have developed delirium and researchers may have been reluctant to test them. It is possible for researchers to be trained to score the CAM based solely on bedside observations; a process that is particularly useful when patients are unwell and poorly responsive. This approach was not applied in the current study, but may be useful for future studies.

Based on the experience from our and other multisite delirium studies [14–17], the following recommendations for training and monitoring are provided for multisite delirium studies (Table 4). These recommendations for initial training and ongoing monitoring and performance checks are made with the intent of assuring high quality, accurate and reliable delirium ratings for research studies. These procedures require an expert delirium assessor at each site who can train and monitor other team members. While each of the individual steps can be adapted to local circumstances, the overall principles of didactic training, individual practice sessions and IRR assessments, including a substantial number of patients with delirium, are critical to achieving high quality delirium assessments. Moreover, ongoing monitoring and performance checks are essential to assure consistent performance over time by all research staff. These include coding sessions with project investigators and key staff from all sites, ongoing IRR (recommended every six months), and training new study staff. The study investigators should build these steps into the overall study design and approach and ensure adequate resources are available to enable this training and ongoing monitoring to occur by the study coordinating centre.

**Table 4:** Recommendations for Confusion Assessment Method training and oversight for multisite studies^a^

Initial training and standardisation	
Didactic overview	• Classroom: guided review of the CAM Training Manual
	• Interactive review and scoring of training videos
Individual practice sessions	• Paired practice interviews with an experienced delirium assessor
	• Mimic 2 patients with and 2 patients without delirium
Pilot interviews with patients	• Experienced delirium assessor observes new research assistant interviewing patients and gives feedback
	• Interview 2 delirium and 2 non-delirium patients
Inter-rater reliability assessments (baseline standardisation)	• Pairs of interviewers observe same patients and score CAM independently
	• After interview, compare and discuss ratings
	• Continue until 100% agreement achieved
	• Minimum of 2 delirium and 5 non-delirium patients
	• Early pairs should include experienced delirium assessor
CAM-only training	• Intended to score cases where patients are poorly responsive or interviews are incomplete
	• Training to code CAM features based on bedside observations. If patients unresponsive, may only be able to code altered level of consciousness
Ongoing monitoring and performance checks	
Coding sessions	• Regularly scheduled meetings to discuss any questions on coding the CAM features
	• Involve project directors and key staff from each study site, and include at least one delirium expert clinician
	• Minimum of 2 times per month (ideally weekly) throughout the study
	• Use sessions as opportunity for retraining
Ongoing inter-rater reliability Assessments (performance checks)	• Local: all staff undergo paired ratings with experienced delirium assessor at the site; ratings compared and discussed. Recommend: every 6 months throughout study
	• Cross-site: One gold-standard expert rater performs spot checks at all study sites with inter-rater assessments at least once per year. Alternative approaches may utilise videoconferencing or face-time for inter-rater assessments across sites.
New staff training	• Complete all steps of initial training when any new staff member joins the study to maintain high quality ratings
	• Verify inter-rater reliability with existing staff

^a^Note: All steps should be overseen by the central coordinating centre, and one fully-trained, experienced delirium assessor (principal investigator, project director, or experienced research staff member) is required at each site to provide ongoing monitoring and training locally. For optimal training, all raters should be trained by an experienced CAM rater

Multicentre clinical trials are not likely to be rigorous or reproducible if outcomes cannot be reliably and accurately assessed across participating sites. While delirium is a common and clinically meaningful outcome, there are currently no biomarkers that confirm or exclude a diagnosis of delirium. Therefore, it is essential in multicentre clinical trials that (i) validated methods are used for clinical delirium detection and (ii) that those assessing for delirium are appropriately trained in the use of the validated detection instrument such that their assessments are demonstrably reliable. Several studies have previously found difficulty in showing both validity and especially reliability in assessing delirium. For example, one study in the emergency department found that structured teaching interventions alone were not sufficient for ensuring either accuracy or IRR in delirium assessment [18]. Even structured training and use of standardised tests has not always produced reliable assessments. This was highlighted in a multicentre study in which experts used the CAM for the Intensive Care Unit as well as the Delirium Rating Scale-Revised-98. Despite this apparently rigorous approach, there was poor agreement between the expert raters [19]. These investigators concluded that it was most important for the ability to conduct multicentre studies with validity that researchers must develop more reliable instruments and training methods for detecting delirium. The CAM has now been shown to be highly reliable in diagnosing delirium when compared against an expert (geriatrician, psychiatrist, or neurologist), using Diagnostic and Statistical Manual of Mental Disorders-based criteria. At the same time, the CAM can be completed in about 5–10 min and can be conducted by scrupulously trained RAs. Another valuable characteristic of the CAM is that it can be used for assessment of delirium severity [7, 20]. The trial by Maybrier et al. [21] was an important antecedent to the current study. It demonstrated, using a different but similarly rigorous training approach, that investigators at multiple international sites, with varying levels of clinical experience, could reliably assess delirium using the CAM.

Members of our research team have previously demonstrated the value of actual patient training complemented by video education [21]. It is likely that the rigour of the training and the reliability of the instrument used (in this case the repeatedly validated CAM) are more important than the training method. Thus both methods (video-based and patient-based) can provide a solid foundation. Ultimately, reliability must be demonstrated with assessment of actual patients in relevant clinical situations.

## Conclusion

This study provides an important contribution towards future multicentre studies and clinical trials of delirium in documenting our approach to standardisation, along with lessons learned. Standardisation of key outcome measures is critical to the quality of any multisite study using multiple assessors. We hope our recommendations for future multisite training and standardisation (Table 4) will assist with future studies of this type. With the application of the training methods described in this study and the recommendations summarised in Table 4, multisite studies should be able to achieve high quality delirium assessments for large numbers of patients with little missing data across even geographically dispersed sites. Following these methodological recommendations will be necessary to achieve scientific rigour and reproducibility. This is especially relevant since multisite studies are being conducted more frequently to improve the understanding and management of delirium. The standardisation of multisite delirium assessment provides a much-needed advance for the field.

## Abbreviations

AMTS: Abbreviated mental test score
CAM: Confusion assessment method
IQR: Interquartile range
IRR: Inter-rater reliability
MotYB: Months of the Year Backwards
NHS: National Health Service
RA: Research assistant
UK: United Kingdom
